# Development and validation of a precise flow injection method for the assessment of brexpiprazole, with application to pharmaceutical dosage forms and human plasma analysis

**DOI:** 10.1186/s13065-024-01240-0

**Published:** 2024-07-26

**Authors:** Sayed M. Derayea, Al Amir S. Zaafan, Dalia M. Nagy, Mohamed Oraby

**Affiliations:** 1 Analytical Chemistry Department, Faculty of Pharmacy, Minia University, Minia, 61519 Egypt; 2 Department of Pharmaceutical Analytical Chemistry, Faculty of Pharmacy, Sohag University, Sohag, 82524 Egypt

**Keywords:** Brexpiprazole, Flow injection analysis, Fluorescence, Pharmaceuticals, Human plasma

## Abstract

A novel antipsychotic medication named brexpiprazole (BRX) is currently employed for the treatment of schizophrenia and other psychotic disorders. Because BRX’s molecular structure includes a benzothiophene ring, it natively fluoresces. To detect BRX with precision and speed, a flow injection-fluorometric method, which is both sensitive and selective, is recommended. The fluorescence detection was conducted at 364 nm following excitation at 326 nm to capture the strong intrinsic fluorescence of BRX. The carrier solution employed was a mixture of phosphate buffer (pH 4, 10 mM) and acetonitrile (50: 50, v/v), with a flow rate of 0.5 mL min^− 1^. The calibration curve, based on peak areas, exhibited linearity within the concentration range of 20–350 ng mL^− 1^, with a remarkable correlation coefficient (r^2^) of 0.9999. The limit of quantitation was 9.7 ng mL^− 1^, and the limit of detection was found to be 3.2 ng mL^− 1^. This method was applied to quantify BRX in Neopression^®^ tablets, achieving recovery within an acceptable range without interference from the tablet’s additives. Additionally, the proposed approach was successfully utilised to quantify the drug in spiked human plasma. The approach underwent validation following ICH requirements.

**Supplementary Information:**

The online version contains supplementary material available at 10.1186/s13065-024-01240-0.

## Introduction

**Fig. 1:**
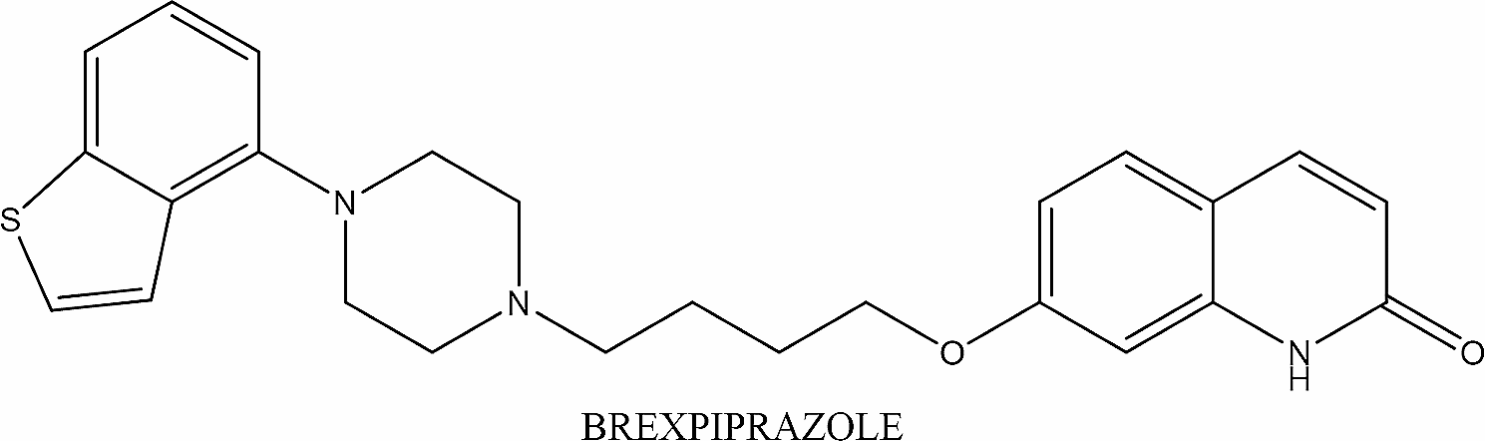
The chemical structure of BRX

Brexpiprazole (BRX), depicted in Fig. 1, is a 7-4-[4-(1- benzothiophene-4-yl) piperazin-1-yl] butoxy-1, 2- dihydroquinoline-2-one. BRX is categorized as an atypical antipsychotic medication. It has been granted approval as a therapeutic agent for schizophrenia and as a complementary therapy for major depressive disorder [1]. The FDA granted its therapeutic use for schizophrenia and depressive disorders in July 2015. BRX exhibits both blocking and activating effects at its target receptor, offering potential benefits in reducing restlessness and agitation due to its predominant blocking and diminished stimulating properties [2]. It also acts by modulating the activity of serotonin and dopamine. Functionally, BRX is considered as a new drug that stimulates partially 1 A serotonin and D2 dopamine receptors, it is also a powerful blocker of serotonin 2 A receptors, and an antagonist of noradrenergic alpha 1B and 2 C receptors [3]. BRX’s higher effectiveness allows for the use of lower doses compared to other antipsychotic drugs. Notably, its affinity for serotonin 5HT1A receptors is notably higher, leading to fewer adverse effects, such as akathisia and extrapyramidal symptoms, when compared to other antipsychotic medication classes [4].

Existing analytical methods for BRX, as revealed in the literature review, encompass spectrophotometric [5–7], spectrofluorimetric [8], HPLC [2, 3, 7, 9–11], TLC [2, 12], and electrochemical method [13]. These approaches require thorough sample preparation, substantial quantities of pure organic solvents, and lead to increased analytical expenses and environmental concerns. Spectrophotometric techniques, though less sensitive, are seldom used in the examination of biological fluids [14]. In contrast, chromatographic methods, while extremely sensitive and suitable for the study of drug degradation products and pharmacokinetics, are hindered by their time-consuming nature, the need for expensive, advanced equipment, significant quantities of exceptionally pure organic solvents, and the requirement for well-trained personnel [15–17].

Consequently, this study aims to introduce a novel approach by developing a flow injection method for BRX quantification, marking the first instance of such a method for BRX measurement. This approach offers a time-saving, sensitive, and solvent-efficient alternative for evaluating BRX in tablet form and spiked human plasma. The procedure validation adheres to the (ICH) recommendations [18]. The methods that using spectrofluorimetric detectors are highly selective due to the presence of two spectra, extremely sensitive, and need very easy sample preparation [19–21]. Flow analysis has become a crucial field in modern analytical chemistry. Its current importance is due to the comprehensive development of specialized instrumentation, its many advantages compared to conventional methods, a wealth of scientific and patent documents, and the abundance of applications tailored to diverse fields such as chemical analysis, food and agricultural chemistry, as well as environmental and clinical chemistry [22].

## Experimental

### Instrumentation

The flow injection setup utilized in this study comprised a Sykam S 1130 HPLC quaternary pump equipped with an optional built-in vacuum degasser (Sykam GmbH, Gewerbering, Germany). Samples were introduced into the system using a Hamilton HPLC syringe (Franklin, MA, USA). To carry out fluorometric measurements, a detector RF-20 A (Shimadzu, Kyoto, Japan) was connected to the HPLC system. It’s important to note that, in this study, the HPLC system’s column was not employed. Aquatron water still A4000D was used to produce double-distilled water (Cole-Parmer, Staffordshire, UK). The pH was regulated with a Jenway 3510 pH meter (Staffordshire, UK). A Mettler Toledo 5-digit balance (Greifensee, Switzerland) was utilized for weighing.

### Materials and chemicals

The BRX pure powder was received as a generous donation from Marcyrl Pharmaceutical Industries (Obour City, Cairo, Egypt). We procured acetonitrile and methanol of analytical quality from Sigma Aldrich (Darmstadt, Germany). The sources for orthophosphoric acid and dipotassium hydrogen phosphate were El Nasr Chemicals Chemical Co (Cairo, Egypt). Additionally, Neopression^®^ 4 mg tablets (Batch no. 2,134,327) were obtained from the Egyptian marketplace. Lastly, Whatman grade No. 1 filter papers (Maidstone, Kent, United Kingdom) were utilized.

### Flow injection conditions

The method was carried out with a carrier solution that was a combination of (50: 50, v/v) acetonitrile and phosphate buffer (pH 4, 10 mM). Prior to use, this mixture was filtered through a 0.22 μm filtration membrane and degassed. The liquid flowed through the system at a flow rate of 0.5 mL min^− 1^. The native fluorescence of BRX was determined at an emission wavelength of 364 nm following excitation at 326 nm. Using an injection volume of 20 µL, the whole procedure was performed at room temperature.

### Preparation of carrier solution

Approximately, 0.871 g of dipotassium hydrogen phosphate was transferred into a 500 mL volumetric flask. Subsequently, 500 mL of double-distilled water was added in the flask, and the mixture was thoroughly shaken. The pH of the resulting solution was modified to reach a value of 4.0 with orthophosphoric acid. Following this, the solution underwent filtration through a 0.22 μm filtration membrane, and any remaining air was removed by subjecting the solution to 5 min of sonication. Finally, it was blended with acetonitrile (50: 50, v/v).

### Preparation of standard stock solution

A 1 mg mL^− 1^ stock solution was formed by precisely measuring 10 mg of pure BRX and dissolving it in 10 mL of methanol. For the BRX working standard solution, the carrier solution was used. The stock solutions were stored in the refrigerator. Subsequently, BRX solutions with concentrations of 20, 50, 100, 150, 200, 250, 300, and 350 ng mL^− 1^ were generated through a series of dilutions using the carrier solution.

### Preparation of pharmaceutical tablets

Ten tablets of Neopression^®^ 4 mg were finely ground. An accurately measured amount of tablet powder, equivalent to 4 mg of BRX, was employed. The BRX was then extracted using analytical-grade methanol in a volumetric flask through sonication lasting 30 min. Subsequently, the volume was adjusted to 50 mL with the same solvent. Whatman grade No. 1 filter paper was utilized to filter the resultant solution, with the initial portion of the filtrate being discarded. For the preparation of the assessed concentration, the extract was diluted using the carrier solution. Five measurements of BRX concentration were determined following the standard analytical procedure. The tablets’ actual content was determined using the corresponding regression equation.

### Preparation of spiked human plasma

Blood samples were collected from the forearm veins of healthy volunteers at Misr Hospital in Sohag, Egypt. These samples were placed in tubes containing heparin. Prior to experimenting, the purpose of the study was explained to each volunteer, and they provided their written consent. The use of plasma specimens from healthy individuals was in accordance with the Helsinki Recommendations [23, 24].

To conduct the experiment, 100 µL of drug-free human plasma was mixed with 100 µL of various concentrations of BRX. This mixture was then treated with 200 µl of acetonitrile to precipitate proteins. The solution was centrifuged for 30 min at 4000 rpm following a powerful 30-second vortex. Clear supernatant samples were taken, and a standard analytical procedure was applied. This methodology was employed to assess the drug under investigation at four different concentrations, with three replicates for each concentration.

## Result and discussion

### Detection wavelength selection

To select an appropriate analytical wavelength, a solution containing 10 µg mL^− 1^ of BRX was prepared by diluting a standard solution. Specifically, 10 mg of standard BRX powder was placed in a 10 mL volumetric flask and dissolved in a small volume of methanol. The flask was then filled up to the mark using the same solvent. From this solution, 0.1 mL was withdrawn and transferred into a separate 10 mL volumetric flask, again brought up to the mark with the same solvent, resulting in a concentration of 10 µg mL^− 1^. This solution was analyzed using a double-beam spectrophotometer in the range of 200 to 400 nm, with methanol as the blank. The resulting spectrum revealed that the maximum absorption wavelength (λ_max_) of BRX was 326 nm, which was chosen as the excitation wavelength for the subsequent analysis. To prepare a lower concentration standard solution of 0.5 µg mL^− 1^ of BRX, methanol was also used to make dilution of the standard solution appropriately. This diluted solution was then examined for its emission wavelength using a spectrofluorometer after excitation at 326 nm. It was determined that BRX exhibited its maximum fluorescence at 364 nm, and this wavelength was selected for the emission analysis.

### Carrier solution selection

**Fig. 2:**
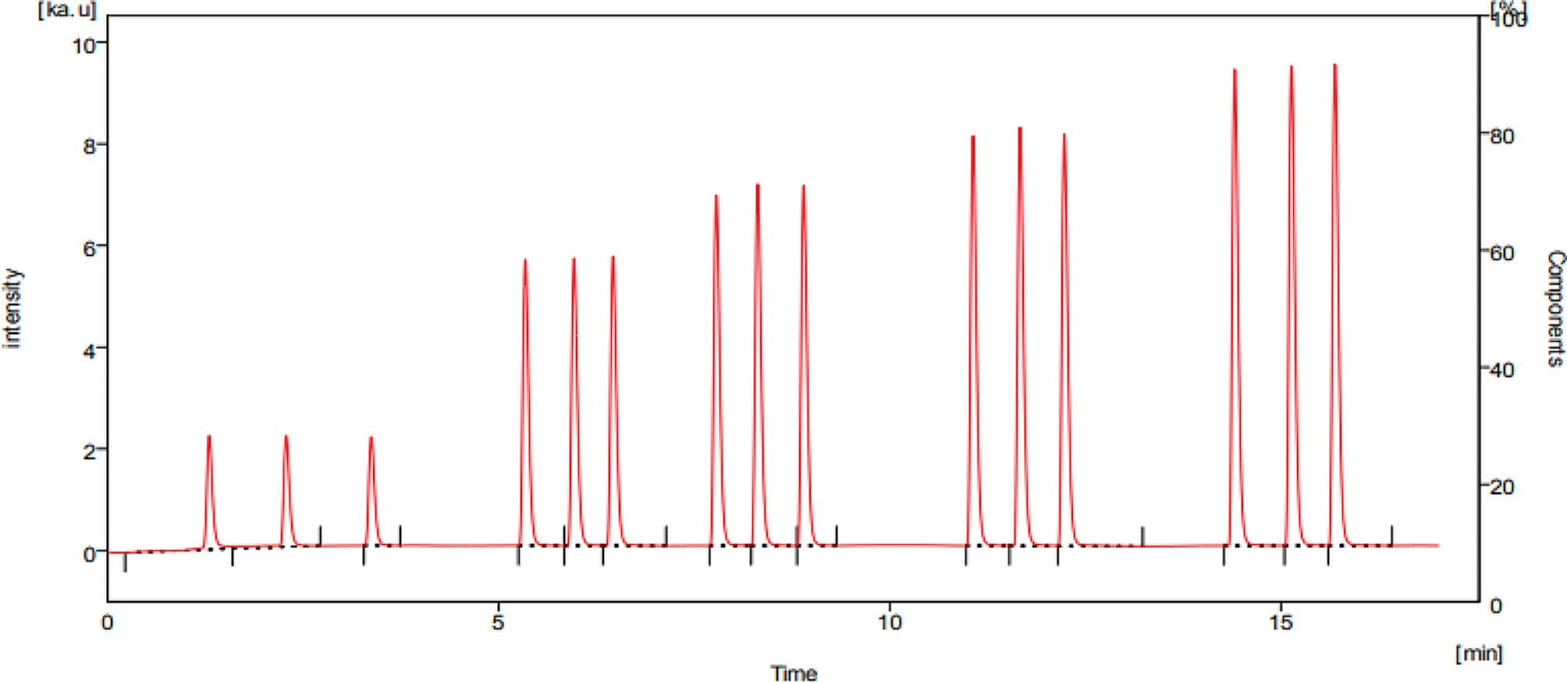
BRX FIAgram 50, 150, 250, 300, and 350 ng mL^-1^

The BRX standard solution was introduced into the equipment and tested with various solvent systems. To determine the best solvent system for BRX, we explored several options, including methanol, acetonitrile, and water in different combinations and pH conditions. The most intense fluorescence occurred when we combined a buffer with acetonitrile. To identify the ideal proportion of these two components for the method, we experimented with various ratios, such as 20:80, 40:60, 50:50, 70:30, 80:20, and 90:10. We discovered that the most effective carrier solution system is a 10 mM phosphate buffer and acetonitrile in a 50:50 volume-to-volume ratio. We also tested different pH levels and found that pH 4 yielded the highest fluorescence (Fig. 2).

### Optimization of the flow rate

The FIA system’s flow rate plays a crucial role as it has a notable impact on the peak area, which reflects the fluorescence intensity response. To optimize the flow rate, various rates (0.5, 0.8, 1, and 1.2 mL min^− 1^) were tested. Interestingly, it was noticed that increasing the flow rate led to a substantial decrease in the peak area. Consequently, 0.5 mL min^− 1^ was determined to be the optimal flow rate, as it yielded the highest peak area with a sharp peak.

### Validation of the suggested method

Based on ICH Q2 (R1) guidelines, the validated analytical procedure [18] was assessed in terms of parameters such as linearity, detection limit (LOD), quantitation limit (LOQ), precision, accuracy, robustness, and similar attributes.

#### Linearity

Several typical solutions of the studied medication were analyzed at different concentrations using the standard analytical procedure. By plotting the peak area against the eventual drug concentration, the calibration curve was established for the drug. The linearity plot for the proposed assay of BRX covered a concentration range of 20–350 ng mL^− 1^, displaying an impressive correlation coefficient (r^2^) of 0.9999. The regression data, calibration data, and correlation coefficients are summarized in Table 1.

**Table 1:** Regression equation and validation parameters for the proposed flow injection method

Parameters	BRX
Linear range (ng mL^− 1^)	20–350
Slope (b)	141.21
Regression Equation	Y = 141.21X + 2767.73
Standard deviation of slope (S_b_)	0.66
Intercept (a)	2767.73
Standard deviation of intercept (S_a_)	137.55
Correlation coefficient (r)	0.9999
Determination coefficient (r^2^)	0.9998
LOD (ng mL^− 1^)	3.21
LOQ (ng mL^− 1^)	9.74

#### Limit of detection and limit of quantitation

The LOD represents the minimum detectable amount within a sample under the specified experimental conditions, although it is not measurable with precision. On the other hand, the LOQ signifies the smallest amount of BRX in a sample which can be measured with a satisfactory level of precision and accuracy. To determine these values, the following formulas were employed: LOD = 3.3 times the standard deviation of the intercept (SD) divided by the slope of the calibration curve (S). LOQ = 10 (SD)/ S. The values for LOD and LOQ can be found in Table 1.

#### Precision

The study examined the precision of BRX, both within the same day (intra-day) and across different days (inter-day), using three concentrations: 100, 200, and 300 ng mL^− 1^. To assess intra-day precision, three consecutive analyses were performed on the same day, whereas inter-day precision was evaluated by performing the analytical procedure over three successive days. This experiment was conducted three times. The recovery values obtained ranged from 98 to 102%, and (% RSD) values were observed to be well within acceptable limits, not exceeding 2.0%. A summary of the results for both intra- and inter-day precisions can be found in Table 2.

**Table 2:** Precision for proposed flow injection method for the determination of BRX

Precision level	Added concentration (ng mL^-1^)	Measured concentration (ng mL^-1^)	Recovery* (%)	Standard deviation	RSD (%)
Intra-day (Day1, *n* = 3)	100	100.69	100.69	1.06	1.05
	200	196.70	98.35	0.14	0.14
	300	297.27	99.09	0.48	0.49
Inter-day (three days, *n* = 9)	100	100.02	100.02	1.33	1.33
	200	199.26	99.63	1.26	1.26
	300	300.60	100.20	0.93	0.93

*Mean of three determinations

*RSD* Relative standard deviation

#### Accuracy

The BRX recovery was determined by employing the standard addition technique to evaluate the method’s precision. Various quantities of the standard solution were introduced into tablet solutions previously analyzed. This recovery assessment encompassed five distinct concentration levels and was conducted in triplicate. The results, including the calculated percentage recoveries and associated SD, are presented in Table 3. The average BRX recoveries ranged from 98 to 102%, and the standard deviations remained below 2.0%. These findings demonstrate that the proposed methodology is sufficiently accurate.

**Table 3:** Evaluation of the accuracy of the analytical procedure by standard addition method (*n* = 3)

Amount taken (ng mL^− 1^)	Amount added (ng mL^− 1^)	Amount found (ng mL^− 1^)	Recovery* (%)	Standard deviation	RSD (%)
100	0.00	99.80	99.80	0.92	0.92
100	50	151.47	100.98	1.11	1.10
100	100	198.40	99.20	1.08	1.09
100	150	252.60	101.04	0.67	0.66
100	200	301.02	100.34	0.32	0.32

*Mean of three determinations

*RSD* Relative standard deviation

#### Robustness

The method’s robustness was evaluated by examining its performance when subjected to minor adjustments in the experimental parameters. These included slight variations in the carrier solution composition (2%), minor fluctuations in flow rate (0.1 mL min^− 1^), changes in pH (0.1), and shifts in the detection wavelength (2 nm). The findings are summarized in Table 4. Since the obtained RSD remained below 2%, it is evident that the method remained largely unaffected by these minor variations in the tested variables. This confirms the robustness of the proposed technique.

**Table 4:** Robustness for 200 ng mL^-1^ of BRX (*n* = 3)

Parameter		Recovery͙͙* (%)	Standard deviation	RSD (%)
Flow rate	0.4	101.51	1.67	1.65
	0.6	98.79	0.62	0.63
Percentage of acetonitrile (%)	48	99.89	0.40	0.40
	52	100.24	1.11	1.11
pH	3.9	101.29	0.80	0.79
	4.1	100.27	0.87	0.87
λ excitation (nm)	324	98.52	0.16	0.17
	328	99.11	0.79	0.80
λ emission (nm)	362	98.66	0.30	0.31
	366	100.22	0.52	0.52

*Mean of three determinations

*RSD* Relative standard deviation

### Applications of the method

#### Application to pharmaceutical dosage forms

**Fig. 3:**
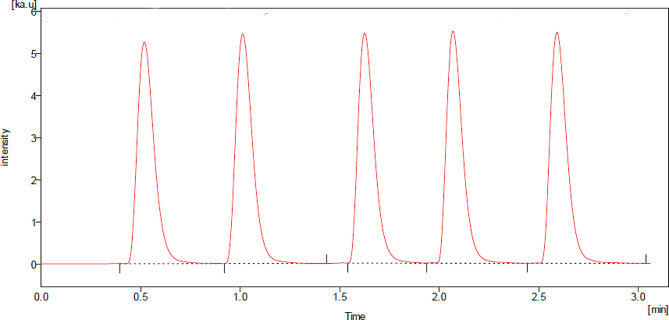
BRX (150 ng mL^-1^) FIAgram in dosage form

An analysis of the drug content in a commercially available dosage form, Neopression^®^ 4 mg tablets, was conducted using a general analytical method, illustrated in Fig. 3. The proposed approach yielded a satisfactory percentage recovery of 98.73 ± 1.42. This notably high percentage recovery also indicated that the tablet’s non-active ingredients did not have a discernible impact. Furthermore, the results obtained through the suggested approach were compared to those from a previously published method [7] in terms of precision and accuracy. The calculated *t*- and F- tests values, at a 95% confidence level, did not exceed the corresponding tabulated values, as demonstrated in Table 5. Hence, no significant disparity was observed between the outcomes of the two methods.

**Table 5:** Analysis of BRX in dosage form (Neopression 4 mg/ tablet) and pure form by reported and proposed methods [7]

Parameters	Dosage form		Pure form	
	Proposed method	Reported method	Proposed method	Reported method
% Recovery ^a^	98.73	100.01	99.46	98.84
Standard deviation	1.419	1.624	1.23	1.38
Variance	2.014	2.637	1.51	1.90
Number of measurements	5	5	5	5
t-test ^b^	1.11	--	0.75	--
F-test ^b^	1.32	--	1.26	--

^a^Mean of five determinations

^b^Tabulated value at 95% confidence limit, F = 6.388 and *t* = 2.306

#### Application to spiked human plasma

**Fig. 4:**
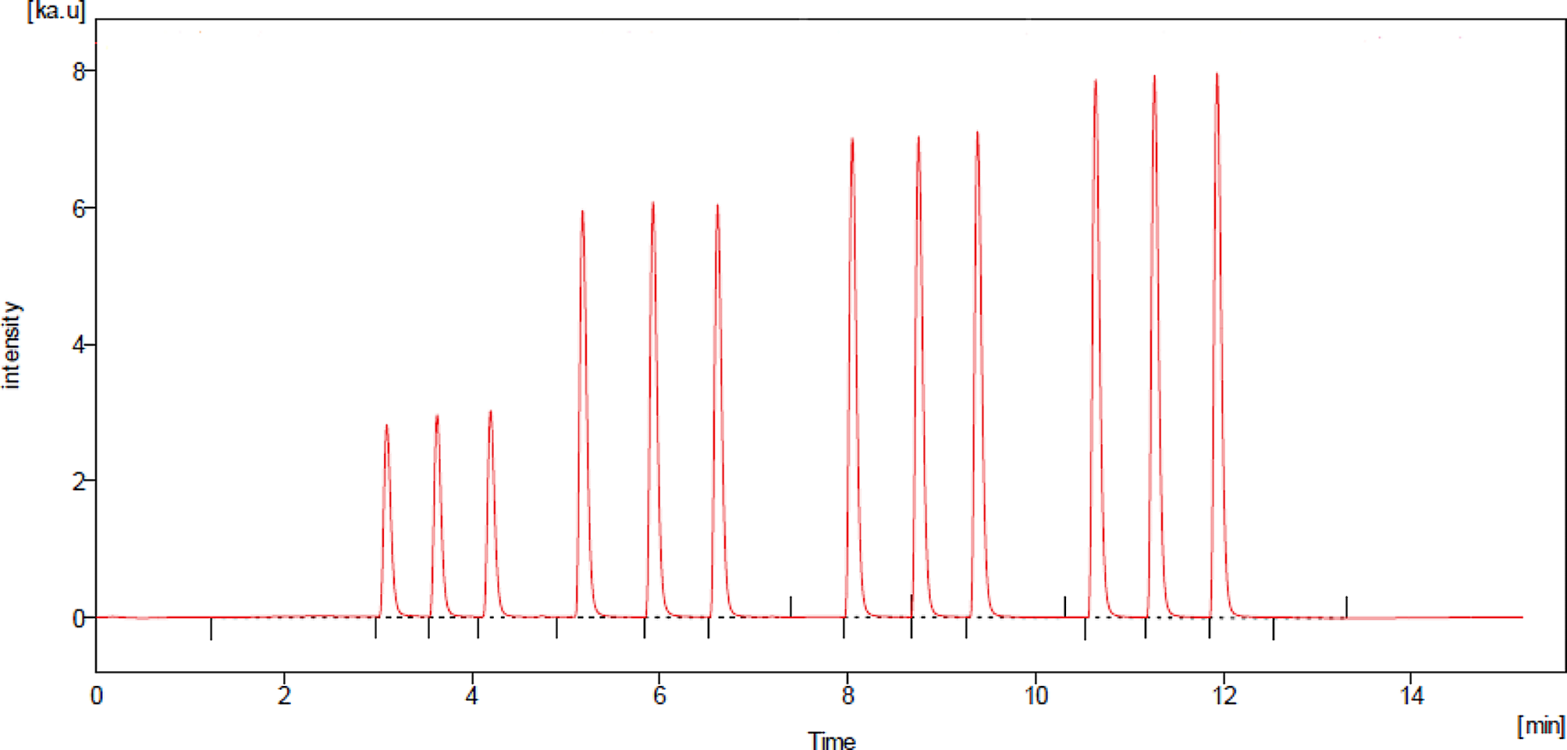
BRX FIAgram in human plasma 50, 100, 200, 300 ng mL^-1^

The highly sensitive FIA method effectively detected BRX in human plasma samples spiked with varying medication concentrations. To achieve this, different drug concentrations were mixed with human plasma, and acetonitrile was introduced to precipitate proteins. Following centrifugation, the recommended analytical protocol was applied. BRX exhibited excellent recovery rates (Fig. 4), and the impact of interfering plasma components was notably reduced, as illustrated in Table 6. The respective drug concentrations were determined by utilizing the resulting regression equation (Y = 141.21X + 2767.73).

**Table 6:** The application of the developed method to measure the drug in spiked human plasma

Drug conc. (ng mL^− 1^)	% Recovery*	SD	%RSD
50	102.53	2.26	2.20
100	100.33	1.11	1.11
200	99.17	1.28	1.29
300	100.98	0.48	0.47

*Mean of three determinations, *SD* standard deviation, *RSD* Relative standard deviation

#### Comparison with the reported methods

A comparison of the analytical performance of the suggested FIA method with other reported methods, including chromatography and spectrometry (Table S1), demonstrates that, the current method has a very low limit of detection (3.21 ng mL^− 1^). Although a few of the previously reported methods had higher sensitivity, the sensitivity obtained from the current work is satisfactory for the quantification of BRX in biological samples and investigating the pharmacokinetic behavior of BRX. The greenness of the proposed method is not very high due to usage of acetonitrile solvent, however, it has higher greenness than reported chromatographic methods that have higher sensitivity [7, 9]. Meanwhile, the current method is characterized by ease of analysis, highly simple and selective procedure. Furthermore, no extensive sample treatment is needed and is cost-effective. All these merits make the present method highly convenient for routine analysis in quality control laboratories.

## Conclusion

This study introduced a new flow injection-fluorometric technique which was utilized to measure BRX in bulk materials, pharmaceutical dosage forms, and human plasma samples that were intentionally spiked with the compound. The suggested approach has been highlighted by its ease of use, sensitivity, precision, and accuracy. It demonstrated selectivity and reproducibility in the analysis of BRX, showing no interference from additives as confirmed through statistical analysis. The procedure effectively quantified BRX in pharmaceutical dosage forms, producing results closely matching the labeled values of commercially available tablets. Moreover, its high sensitivity makes it suitable for analyzing biological samples and quantifying BRX in spiked human plasma. The method’s validity was confirmed by ICH guidelines.

### Electronic supplementary material

Below is the link to the electronic supplementary material.

Supplementary Material 1

## Data Availability

The datasets used and/or analyzed during the current study are available from the corresponding author on reasonable request.

## References

[1] Kumar VG, Mondal S. A new stability indicating RP-HPLC method for estimation of brexpiprazole. J Drug Deliv Ther. 2019;9:214–22.

[2] Thakkar AM, Chhalotiya UK, Parekh N, Desai JV, Shah DA. Stability Indicating TLC Method for Quantification of Brexpiprazole in Bulk and its Pharmaceutical Dosage Form and determination of Content Uniformity. J Chromatogr Sci. 2019;57:644–52. 31095672 10.1093/chromsci/bmz039

[3] Sravani A, Naga Durga C, Divya U, Suneetha C, Suresh P, Tirumaleswara Rao B, et al. Method development and validation for the estimation of brexpiprazole in drug substance by RP-HPLC method. Indo Am J Pharm Res. 2017;7:8560–4.

[4] Gosar A, Phadke R. Gradient high performance liquid chromatography method for determination of related substances in brexpiprazole API. Int J Dev Res. 2018;8:21416–24.

[5] Patel P, Mashru R, Novel. UV spectrophotometric & chemometrics assisted spectrophotometric methods for simultaneous estimation of Brexpiprazole and Sertraline: a statistical analysis. Pharma Innov J. 2020;9:29–42.

[6] Patel P, Mashru R. Design, optimization, and validation of chemometrics assisted spectrophotometric methods for simultaneous determination of brexpiprazole and aripiprazole. Pharma Innov J. 2020;9:258–65.

[7] Thakkar AM, Chhalotiya UK, Parekh N, Desai JV, Dalwadi HB, Shah DA. Quantification of brexpiprazole in bulk and its pharmaceutical dosage form by UV–visible spectroscopic and SIAM RP-HPLC method. Austin Chromatogr. 2018;5:1050–6.

[8] Derayea SM, Zaafan AAS, Nagi DA, Oraby M. Augmentation of Brexpiprazole fluorescence through photoinduced electron transfer inhibition for the sensitive spectrofluorimetric assay of pharmaceutical dosage forms and spiked human plasma: application to content uniformity testing. Spectrochim Acta Part Mol Biomol Spectrosc. 2023;301:122948. 10.1016/j.saa.2023.122948 37285746

[9] Bhawar HS, Thete S, Shinde GS. Development and validation of stability indicating RP-HPLC method for estimation of brexpiprazole from bulk and tablet form. J Drug Deliv Ther. 2019;9:141–5.

[10] Gosar A, Rajendra P. Gradient high performance liquid chromatography method for determination of related substances in brexpiprazole API. Int J Dev Res. 2018;8:21416–24.

[11] Vanaplli GK, Mondal S. A new stability indicating RP-HPLC method for estimation of brexpiprazole. J Drug Deliv Ther. 2019;9:214–22.

[12] Vahora S, Chhalotiya UK, Kachhiya H, Tandel J, Shah D. Simultaneous quantification of brexpiprazole and sertraline HCl in synthetic mixture by thin-layer chromatography method. J Planar Chromatogr - Mod TLC. 2021;34:549–57. 10.1007/s00764-021-00142-4

[13] Salama FM, Attia KAM, El-Shal MA, Said RAM, El-Olemy A, Abdel-Raoof AM. Anodic stripping voltammetric methods for determination of brexpiprazole and its electrochemical oxidation behavior in pure form and pharmaceutical preparations. J New Mater Electrochem Syst. 2019;22:91–7. 10.14447/jnmes.v22i2.a05

[14] Oraby M, Abdelhamid AA, Mohamed KMH, Mehanni AHE, Elsutohy MM. Rapid and simple Spectrophotometric Method for the determination of antiviral and anti-parkinsonism drugs. J Appl Spectrosc. 2020;87:289–95. 10.1007/s10812-020-00998-0

[15] Hassan MG, Ikeda R, Wada M, Kuroda N, Abdel-Wadood HM, Mohamed HA, et al. Interaction study of acetylcholinestrase inhibitors on pharmacokinetics of memantine in rat plasma by HPLC-fluorescence method. Biomed Chromatogr. 2013;27:1685–9. 23861199 10.1002/bmc.2980

[16] Khorshed AA, Elsutohy MM, Mohamed AA, Oraby M. HPTLC Method for the Ultrasensitive Detection of Triamterene in plasma. J Chromatogr Sci. 2022;60:267–73. 34128052 10.1093/chromsci/bmab076

[17] Derayea SM, Elhamdy HA, Oraby M, El-Din KMB. Simultaneous measurement of duloxetine hydrochloride and avanafil at dual-wavelength using novel ecologically friendly TLC-densitometric method: application to synthetic mixture and spiked human plasma with evaluation of greenness and blueness. BMC Chem. 2024;18:1–13. 38702832 10.1186/s13065-024-01195-2 PMC11067093

[18] Requirements for registration of pharmaceuticals for. Human ICH harmonised tripartite guideline validation of analytical procedures: parent Guideline. Text on Validation of Analytical Procedures; 2005.

[19] Zaafan AAS, Derayea SM, Nagy DM, Oraby M. Evaluation of the on–off fluorescence method for facile measurement of vilazodone in pharmaceutical dosage form; application to content uniformity testing and greenness evaluation. Spectrochim Acta - Part Mol Biomol Spectrosc. 2024;319:124519. 10.1016/j.saa.2024.124519 38815314

[20] Derayea SM, Elhamdy HA, El-Din KMB, Oraby M. Versatile applications of a spectrofluorimetric approach based on photo-induced electron transfer blocking of Lurasidone. J Mol Liq. 2023;391:123264. 10.1016/j.molliq.2023.123264

[21] Derayea SM, Oraby M, Zaafan AAS, Hamad AA, Nagy DM. A facile on – off fluorescence approach for fluvoxamine determination in pharmaceutical tablets; application to content uniformity testing. 2024;14:8283–92. 10.1039/d3ra08257a PMC10926349 38469194

[22] Trojanowicz M, Kołacińska K. Recent advances in flow injection analysis. Analyst. 2016;141:2085–139. 26906258 10.1039/C5AN02522B

[23] Rits IA. Declaration of Helsinki. Recommendations guidings doctors in clinical research. World Med J. 1964;11:281. 14182999

[24] Derayea SM, Elhamdy HA, El-Din KMB, Oraby M. Novel spectrofluorometric approach for assessing vilazodone by blocking photoinduced electron transfer: analytical performance, and greenness-blueness evaluation. RSC Adv. 2024;14:4065–73. 38288155 10.1039/D3RA08034J PMC10823494

