# Supplementary material for: Development and validation of a precise flow injection method for the assessment of brexpiprazole, with application to pharmaceutical dosage forms and human plasma analysis
Source: BMC Chem. 2024 Jul 26;18(1):137. doi: 10.1186/s13065-024-01240-0 (PMC11282860; doi:10.1186/s13065-024-01240-0)
Supplement: Supplementary file 1 — Supplementary Material 1 [file 13065_2024_1240_MOESM1_ESM.docx]

S1 (Table): Comparison between the proposed and reported methods for the analysis of BRX.

| **Method** | **Matrix** | **Linear range (µg/mL)** | **LOD (µg/mL)** | **Detection** | **Ref.** |
| --- | --- | --- | --- | --- | --- |
| HPLC | Forced degraded samples | 0.1–250 * | 0.02 | UV at 214 nm | [1] |
| HPLC | Forced degraded samples | 10–50 | 0.55 | UV at 215 nm | [2] |
| HPLC (C18) | Bulk powder | 50 - 150 | 3 | UV at 315 nm | [3] |
| HPLC (C18) | induced oxidative degraded sample | 20-100 | 4.77 | UV at 259 nm | [4] |
| HPLC | Forced degraded samples | 10 - 60 | 0.30 | UV at 213 nm | [5] |
| HPLC | Tablets | 5 - 75 | 1.23 | UV at 216 nm | [6] |
| HPLC | Spiked human plasma | 0.01 – 0.4 | 0.003 | UV at 215 nm | [7] |
| HPLC | Tablets | 5 - 75 | 0.64 | UV at 216 nm | [8] |
| UPLC | Invitro dissolution | 20 – 61 | -- | UV at 215 nm | [9] |
| TLC | Tablets | 20 – 160 | 6.6 | UV at 215 nm | [10] |
| TLC | Bulk powder | 15 - 50 | 5.9 | UV at 254 nm | [11] |
| Spectrophotometry chemometrics | Synthetic mixtures | 1 – 7 | 0.19 | UV at 325 nm | [12] |
| Spectrophotometry chemometrics | Bulk powder | 1 – 5 | -- | UV at 325 nm | [13] |
| HPLC | Tablets | 0.01 - 10 | 0.003 | UV at 215 nm | [14] |
| Spectrofluorimetry | Tablets and spiked human plasma | 0.005-0.22 | 0.0008 | Ex at 333 nm & Em at 390 nm | [15] |

* Strange range for HPLC method.
